# Emerging trends and patterns of self-reported morbidity in India: Evidence from three rounds of national sample survey

**DOI:** 10.1186/s41043-017-0109-x

**Published:** 2017-08-09

**Authors:** Kalosona Paul, Jayakant Singh

**Affiliations:** 1 School of Development Studies, Tata Institute of Social Sciences, Opp. Deonar Depot, Mumbai, 400088 India; 2 School of Health Systems Studies, Tata Institute of Social Sciences, Opp. Deonar Depot, Mumbai, 400088 India

**Keywords:** Self-reported morbidity, Disease burden, India

## Abstract

**Background:**

India is rapidly undergoing an epidemiological transition with a sudden change in the disease profile of its population. It is important to understand the changing nature of the burden of disease across the states of India for adequate policy intervention.

**Methods:**

We analyzed the trend and pattern of self-reported morbidity across states of India using three rounds of (52nd, 60th and 71st) National Sample Survey Organization (NSSO) data. Descriptive analysis was carried out to understand the prevalence of self-reported morbidity variation over a period of two decades (1995-2014) and multivariate analysis was performed to identify the significant determinants of various types of self-reported morbidities.

**Results:**

The results indicated an increasing trend of infectious disease, Cardio Vascular Diseases (CVDs) and Non-Communicable Diseases (NCDs) over the last two decades (1995-2014). CVDs increased by a whopping eight-fold and the NCDs increased by three times during this period. A higher prevalence of self-reported morbidity was observed among the elderly and female, particularly in the urban locality. The growing incidence of CVDs and NCDs, especially among the elderly were reported from Kerala, Tamil Nadu, Punjab and West Bengal.

**Conclusions:**

The already constrained public health system in India is likely to face serious challenges with a double burden of communicable and non-communicable diseases. An effective and responsive public health system needs to be in place to make health care services available for NCDs and CVDs at the primary level. In order to ameliorate caregiving, the involvement of family will be critical. Informing the people inculcate healthy habits may be an effective health promotion measure.

## Introduction

India has some of the palpable health indicators in the world. The improvement in infant mortality rate (IMR) and maternal mortality ratio (MMR) in India are awfully slow. The recent sample registration system bulletin reveals that over the last two decades (1990-2015) IMR in India reduced from 88 to only 37 per 1000 live births. The subcontinent of India reports one of the highest MMR i.e. 167 deaths per 100,000 live births [1]. Similarly, life expectancy at birth which is considered as a summary indicator of health and well-being showed only a marginal improvement, an increase of 3 years from 65 years to 68 years during a period from 2001-2011 [2–5]. The health care delivery system of India is characterised by a massive out of pocket expenditure. Government spending on health sector in India is meagre. The public spending on the health in India is less than one percent of the GDP, much lower than many of the African countries [3, 6, 7]. A recent estimate suggests that out of pocket expenditure was nearly 846 billion rupees in 2004 which was about 3.3 percent equal to that of the GDP of the year [8]. In all, evidence suggests somewhat poor health outcomes in India.

Different regions of India experience dissimilar temperature, rainfall and other geographic conditions due to considerable latitude and longitude extensions ranging from the north to south and the east to west. Across the country, there are different set of cultural beliefs and practices that have a significant bearing on the ways the population perceive health. Non-communicable diseases like cardiovascular diseases, cancer, diabetes, chronic obstructive disease, mental disorder and injuries account for about half of all deaths in India [5]. According to the global health observatory report (2012), out of 68 million total deaths globally, an estimated 38.5 million deaths occurred due to NCDs. India is doubly burdened with both communicable as well as non-communicable disease. Although CVDs and other non-communicable diseases are on the rise, communicable diseases continue to be a major public health problem in India [5, 9, 10]. An incessant increase in the communicable disease, CVDs, NCDs has overburdened the already inadequate health systems in India [11, 12]. Studies reveal that the infectious diseases, rapid rising of CVDs and NCDs are attributed mainly due to change in intake of food pattern, urban sprawl lifestyle, poverty, poor quality water supply and unhygienic environment, pollution, etc. [4, 13, 14]. However, the risk of such diseases among the population with different background characteristics is a relatively lesser known fact. Particularly, the socio-economic determinants contributing to the health condition of a population hold significant relevance to inform policy and programme better.

The morbidity pattern of a population is considered as a proxy measure to understand their health status [3, 14, 15]. Measures of self-reported morbidity are directly linked to the health status of any given population. However, limited studies explored the pattern of morbidity across the major states in India using nationally representative large-scale survey data. On the other hand, little information is available about the changing pattern of morbidity prevalence in India from a recent population-based survey. This paper examined the morbidity pattern in India and states in the last two decades based on the International Classification of Disease (ICD), WHO 2012. Promptly, this study investigated the ways in which different self-reported morbidities are associated with factors such as sex, place of residence, level of education, age, monthly per capita consumer expenditure (MPCE), household size, marital status, etc. over two decades to understand the trend and pattern of morbidity.

## Data and Methods

### Data

Three rounds of National Sample Survey Organisation (NSSO) conducted in 1994-95 (52^nd^), 2004 (60^th^) and 2014 (71^st^) respectively were used to examine the morbidity pattern. The NSSO was set up in 1950 as a permanent survey organization by the Ministry of Statistics and Program Implementation to collect data on various facets of the Indian economy through nationwide sample surveys in order to assist in socioeconomic planning and policymaking. Besides gathering information on its core areas, that is, household consumption and expenditure, the NSSO collects detailed information on morbidity patterns of the population from the selected households. Using a multi-stage sampling design, the NSSO covers all the states and union territories in India. It adopts a uniform sampling procedure and geographical coverage; thus, all its rounds of surveys are comparable. The latest round (71^st^) of NSS was titled as ‘India - Social Consumption: Health’. The NSS 60^th^ round survey was based on ‘Morbidity and Health Care’ and the 52^nd^ round was on ‘Survey on Health Care’.

### Sub round information (Table 1)

#### Sampling design

The 52^nd^ NSS morbidity round adopted a stratified two-stage sampling design and the data was collected during 1995-96. The first-stage units were based on the complete enumeration of census villages in the rural area (panchayat wards in case of Kerala) and the NSSO urban frame survey (UFS) blocks for sampling in urban areas. The second-stage units were households in both the sectors. In contrast, a stratified multi-stage design was adopted for both 60^th^ round (2004) and 71^st^ round (2014) survey. The first stage units (FSU) were based on 1991 census villages in the rural sector and UFS blocks for urban sector. The ultimate stage units (USU) were households in both sectors. In the case of large FSUs, one intermediate stage of sampling was selected of two hamlet-groups (hgs)/ sub-blocks (sbs) from each rural/ urban FSU.

**Table 1:** Sub-round information

Sub-Rounds	1995-96	2004	2014
sub-round 1	July - September 1995	January - March 2004	January - March 2014
sub-round 2	October - December 1995	April - June 2004	April - June 2014
sub-round 3	January - March 1996	**-**	**-**
sub-round 4	April - June 1996	**-**	**-**

Sources: NSSO report, 52^nd^, 60^th^ and 71^st^ round

#### Sample size

The information was collected from a total of 120,942 (rural: 71284 and urban: 49658) 73,868 (rural: 47302 and urban: 26566), and 65,932 households (rural: 36,480 and urban: 29,452) in the 52^nd^, 60^th^ and 71^st^ rounds respectively. The data collection period for the 52^nd^ round was spread from July 1995 to June 1996 in four sub-rounds, each comprising three months. In the 60^th^ round, the survey was conducted in two sub-rounds for a duration of three months each from January to June 2004. In the 71^st^ round, the data collection was conducted from January to June 2014 (Table 1).

#### Classification of self-reported morbidity

Information was gathered on 58, 42 and 61 kinds of different morbidities in the 52^nd,^ 60^th^ and 71^st^ rounds respectively. Self-reported morbidities were classified into five broad categories: infectious diseases, cardiovascular diseases (CVDs), non-communicable diseases (NCDs), disability and other disease (Appendix). The disease classification was based on the International Classification of Disease (WHO, 2012). The prevalence of self-reported morbidity was calculated based on the available information on any person who had fallen ill during the 15 days preceding the survey.

#### Statistical analysis

Prevalence of morbidity was calculated per 1000 population. The following formula was used to calculate morbidity prevalence.

$$ \boldsymbol{Mi}=\frac{\boldsymbol{Ai}}{\boldsymbol{Pi}}\ast \mathbf{1000} $$

Where,

Ai= No. of ailing persons

Pi= Total number of persons alive in the sample households

We carried out bivariate analysis between the background characteristics and the outcome variable i.e. morbidities such as infectious disease, CVDs, NCDs, disability and other disease. In the second part of analysis binary logistic regression analysis were performed.The morbidity variable was a dichotomous variable (yes/no). The trend of the self-reported morbidities is presented by sex, place of residence, age, level of education, social group, caste, religion, monthly per capita consumer expenditure (MPCE), marital status and regions in India. And these variables were fitted in the logistic regression model to check its independent effect on each of the morbidity pattern examined.

The equation of logistic regression was the following:

$$ \mathbf{Logit}\left(\mathbf{Y}\right)=\mathbf{\ln}\left(\frac{p}{1-p}\right)=\boldsymbol{\alpha} +{\boldsymbol{\beta}}_{\mathbf{1}}{\boldsymbol{x}}_{\mathbf{1}}+{\boldsymbol{\beta}}_{\mathbf{2}}{\boldsymbol{x}}_{\mathbf{2}}+\in $$

Where, p is the probability of the event and α is intercept, β_s_ are regression coefficients, x_i_ is set of predictors and є is an error term. STATA 12 was used to analyze data.

## Results

### Trends in self-reported morbidity in India

**Fig. 1:**
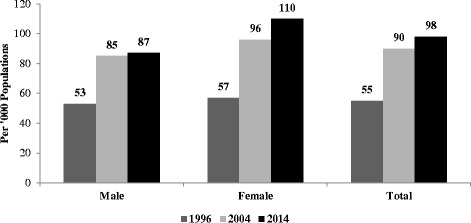
Trends of self-reported morbidity prevalence rate by sex in India, 1995-2014

**Fig. 2:**
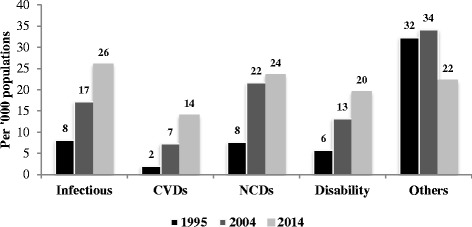
Prevalence of various types self-reported morbidity in India, 1995-2014

The prevalence of self-reported morbidity nearly doubled from 55 to 98 per 1000 populations within a period of two decades i.e. 1995-2014 (Fig. 1). Self-reported morbidity substantially increased in both male and female population. However, the increase was steadily higher among females as compared to males. Infectious disease, CVDs, NCDs, and disability increased drastically within a period of two decades, of which, CVDs increased by seven times, disability increased by four times and both infectious diseases as well as NCDs increased by nearly three times (Fig. 2). However, other types of self-reported morbidities decreased from 32 per 1000 populations to 22 per 1000 from 1995 to 2014 (Table 2).

**Table 2:** Prevalence of different type of self-reported morbidity in India, 1995-2014 (Per ’000 populations)

States & UTs	Infectious			CVDs			NCDs			Disability			Others		
	1995	2004	2014	1995	2004	2014	1995	2004	2014	1995	2004	2014	1995	2004	2014
Andhra Pradesh	5	9	28	2	13	38	9	25	40	12	20	32	37	36	23
Andaman & Nicobar	5	9	42	0	6	39	2	14	56	1	9	41	17	22	22
Arunachal Pradesh	3	31	35	0	1	2	4	4	14	1	5	11	19	10	26
Assam	22	28	13	1	4	1	8	9	3	5	8	6	47	33	10
Bihar	6	14	21	1	2	3	6	9	10	5	5	12	19	23	14
Chandigarh	4	4	16	6	15	16	13	14	28	7	2	35	107	34	48
Chhattisgarh		12	15		2	5		15	7		8	4		33	10
Dadra & Nagar Haveli	9	2	11	0	0	13	1	8	28	12	0	41	36	6	18
Daman & Diu	22	6	27	4	4	68	3	9	64	5	1	27	10	5	12
Delhi	6	1	17	1	2	2	7	3	5	6	2	4	22	6	11
Goa	2	11	74	3	8	34	7	81	56	10	6	2	21	20	15
Gujarat	8	17	33	2	9	19	6	18	23	4	11	19	23	19	10
Haryana	7	17	19	2	8	5	10	29	12	6	12	8	36	31	22
Himachal Pradesh	14	14	17	5	9	12	17	22	17	13	16	25	49	24	12
Jammu & Kashmir	9	11	14	1	3	13	9	25	6	6	13	20	28	19	7
Jharkhand		8	15		1	2		7	19		2	13		17	15
Karnataka	6	8	20	1	6	19	7	17	20	6	12	19	24	23	22
Kerala	8	21	44	9	32	84	18	86	109	13	38	69	63	89	79
Lakshadweep	3	11	45	2	16	55	19	46	72	4	20	43	27	36	33
Madhya Pradesh	5	15	15	1	3	6	4	11	11	2	9	8	28	25	19
Maharashtra	6	17	24	2	11	13	7	26	14	7	22	13	30	33	13
Manipur	0	6	9	1	1	3	2	5	3	0	2	2	4	14	3
Meghalaya	10	23	14	0	1	0	4	1	5	2	10	4	18	15	8
Mizoram	0	7	9	0	0	0	5	2	6	1	2	6	9	4	7
Nagaland	6	31	9	1	0	0	7	2	0	3	3	4	19	22	15
Orissa	6	19	29	1	2	10	4	10	11	4	7	19	48	37	35
Pondicherry	7	23	49	0	29	55	8	35	78	8	48	31	53	57	42
Punjab	7	19	32	6	13	29	14	40	37	9	18	31	44	39	40
Rajasthan	5	12	17	1	3	2	3	15	20	2	8	10	18	23	14
Sikkim	4	10	7	0	1	5	6	23	3	3	7	13	23	9	12
Tamil Nadu	6	13	28	3	9	33	8	25	59	5	15	29	32	37	32
Tripura	21	36	12	2	5	4	9	53	3	7	4	6	76	22	15
Uttar Pradesh	13	26	22	1	4	5	8	19	13	4	11	15	37	42	19
Uttarakhand		12	25		6	2		5	18		10	9		24	32
West Bengal	12	26	45	4	12	19	11	30	36	6	17	40	34	45	41
India	8	17	26	2	7	14	8	22	24	6	13	20	32	34	22

Sources: NSSO Data, 52^nd^, 60^th^ & 71^st^ round,

### Emerging trends of disease pattern across states in India

Assam reported the highest prevalence of infectious diseases in the first two consecutive rounds (22 and 28 per 1000 population respectively) from among the major states in India. However, in the last round of NSS, Assam reported a lower level of prevalence (13 per 1000 population). Similarly, West Bengal reported nearly double the prevalence of infectious diseases from 2004 to 2014 (26 to 45 per 1000 population), and it was the highest from among major states of India in the last round of NSS. Morbidities related to infections more than doubled from 2004 to 2014 (21 to 44 per 1000 population) in Kerala. Over the period of various NSS rounds, self-reported infectious diseases increased drastically, and this increase was the highest in Goa, a whopping 74 per 1000 population from only two per 1000 within a period of two decades. The majority of the north-eastern states, Madhya Pradesh and Uttar Pradesh, showed a decreasing trend in infectious diseases in the last round of NSS. Overall, infectious diseases in India increased from 8 to 26 per 1000 in the last two decades.

Kerala showed an increasing trend in CVDs in all the three rounds of NSS. The CVD prevalence of 84 per 1000 population in 2014 was a massive ten times higher than 1995. Kerala consistently remained as the leading state in self-reported morbidity for CVDs across the three rounds of NSS. Punjab and West Bengal also reported a very high level of CVDs in the first two rounds of NSS. However, undivided Andhra Pradesh and Tamil Nadu surpassed Punjab and West Bengal in the prevalence of self-reported CVDs in the last round of NSS. All the South Indian states including Karnataka were the leading states reporting a higher prevalence of CVDs as compared to other major states in the recent round of NSS. The states such as Bihar, Madhya Pradesh, Rajasthan, Uttar Pradesh, Jharkhand, Chhattisgarh, Assam and Odisha reported a very low level of CVDs across all the three rounds of NSS. However, Odisha reported five times higher CVDs in the recent rounds as compared to the previous round (from 2 to 10 per 1000 population). In all, CVDs doubled in India from 2004 to 2010 with a substantial portion of it being reported from South India.

NCD remained higher in Kerala across all the three rounds. NCDs in Kerala increased by more than six times within a span of two decades. Although Punjab reported consistently higher NCDs in the first two rounds, NCDs marginally decreased in Punjab during the last round of NSS. On the other hand, the prevalence of NCDs in Tamil Nadu increased by seven times, and in undivided Andhra Pradesh, it increased by four times, placing these two states just behind Kerala. West Bengal, Gujarat and Rajasthan also showed an increasing trend in NCDs across the three rounds of NSS. The states such as Assam, Bihar, Chhattisgarh, Delhi, Madhya Pradesh and Odisha indicated a very low level of NCDs prevalence across the three rounds. On the other hand, Haryana, Himachal Pradesh, Jammu and Kashmir, Maharashtra and Uttar Pradesh were a few states where the prevalence of NCDs decreased in the last round of NSS in spite of showing an increasing trend in the second round of NSS.

The majority of the states indicated an increasing trend in reporting morbidity related to disability, of which Kerala, Andhra Pradesh, West Bengal, Punjab, Himachal Pradesh and Tamil Nadu reported higher levels of disability-related morbidity. Similarly, Gujarat, Jammu and Kashmir, Karnataka, Odisha showed a gradual increasing trend in disability related morbidity. On the other hand, Assam, Chhattisgarh, Delhi, Haryana, Madhya Pradesh reported a very low level of morbidity due to disability. Interestingly, other morbidities were also higher in Kerala followed by West Bengal, Punjab, Odisha, etc. The states such as Delhi, Assam and Rajasthan reported slightly lower levels of other morbidity.

### Self-reported morbidity by background characteristics in India 1995-2014

Table 3 provides an overview of the self-reported morbidity by selected background characteristics. Self-reported morbidity was persistently higher among the female population as compared to male population irrespective of the types of morbidities reported. However, the difference between male and female was substantial in the last round of NSS, particularly in reporting disability (14 per 1000 among males versus 25 among females). Urban residents reported a higher prevalence of self-reported morbidity as compared to their rural counterparts for most of the morbidities. Infectious disease was slightly higher in rural areas during the first two rounds of NSS. However, infectious dieases marginally increased among the urban residents in the last round of NSS. CVDs and NCDs were consistently higher among both rural and urban residents. CVDs among the urban population was more than twice likely than their rural counterparts in all the three rounds of NSS. However, NCDs marginally decreased among the rural population in the last round of NSS (20 per 1000 population to 18 per 1000 population). Morbidity related to disability and other morbidity did not indicate much difference between the urban and rural population.

**Table 3:** Prevalence of morbidities by background characteristics in India, 1995-2014 (Per ‘000 populations)

Background Characteristics	Infectious			CVDs			NCDs			Disability			Others		
	1995	2004	2014	1995	2004	2014	1995	2004	2014	1995	2004	2014	1995	2004	2014
*Sex*															
Male	8	17	23	2	6	13	7	21	21	6	12	14	31	31	21
Female	8	18	26	2	8	17	8	22	26	6	14	25	34	37	23
*Place of Residence*															
Rural	9	18	24	2	5	11	7	20	18	6	13	19	33	34	22
Urban	7	14	25	3	15	24	9	27	36	5	15	21	30	33	24
*Education*															
Illiterate	10	23	33	2	6	16	9	24	25	7	16	27	36	43	28
Primary	8	14	22	2	7	14	6	19	21	5	11	17	30	30	23
Higher Secondary	6	12	20	3	10	14	7	22	23	5	11	15	27	26	18
graduate & above	5	10	18	4	17	18	11	25	30	5	10	15	22	22	14
*Age Group*															
<15	8	19	27	0.1	0.2	2	5	12	8	3	4	4	34	39	31
15-34	6	10	17	1	1	2	5	11	8	3	6	9	24	23	15
35-59	9	20	27	4	12	22	10	27	38	8	16	31	34	35	20
60+	21	35	40	15	57	94	37	104	98	36	88	83	67	60	31
*Castes*															
ST/SC	8	18	24	1	3	10	6	17	17	5	11	17	31	32	21
other backward class	NA	16	24	NA	6	15	NA	20	24	NA	13	19	NA	35	23
Other	8	19	26	2	13	21	8	28	29	6	17	23	33	35	23
*Religion*															
Hindu	NA	17	25	NA	7	14	NA	21	23	NA	13	19	NA	34	21
Muslim	NA	20	22	NA	8	14	NA	23	22	NA	13	19	NA	39	25
Christianity	NA	18	33	NA	22	33	NA	51	63	NA	28	30	NA	45	39
Others	NA	19	25	NA	14	22	NA	30	29	NA	17	26	NA	28	28
*Marital Status*															
Never Married	7	16	23	0.3	1	2	5	12	7	3	5	6	31	34	25
Currently Married	9	17	25	3	12	21	9	27	33	7	17	26	32	32	19
Widowed/div/separate	13	32	37	9	35	69	25	69	74	22	64	79	54	58	28
*Wealth Quintile*															
poorest	8	19	26	1	4	11	7	18	17	7	15	21	34	39	23
Poor	8	18	25	1	4	11	7	17	17	5	12	19	33	32	24
medium	8	16	25	2	5	14	7	20	25	6	12	18	33	36	21
Rich	8	18	24	2	8	15	7	23	26	6	11	20	32	34	20
richest	8	16	24	4	16	23	10	30	36	6	16	20	30	31	22
*NSS Region*															
North region	11	22	22	2	5	7	9	21	15	5	12	16	37	37	21
Central region	5	14	15	1	3	6	4	12	10	2	9	7	28	27	16
East region	8	19	29	2	6	10	7	17	20	5	9	22	30	33	26
West region	6	15	24	2	8	11	6	21	18	5	16	14	25	27	12
South region	6	12	29	3	13	38	9	33	50	9	19	33	36	41	33
North-East region	19	27	13	1	4	1	7	12	3	4	7	6	45	28	10

Sources: NSSO Sources: NSSO Data, 52^nd^, 60^th^ & 71^st^ round,

Infectious disease decreased as the level of education increased in all the three rounds of NSS. Conversely, CVDs were higher among the population with a higher level of education across the three rounds of NSS. It is interesting to note that the prevalence of NCDs was higher among both populations with no education and among those who were graduate and above. On the other hand, disability was higher among population with no education. Other morbidities decreased with the level of education across all the three rounds of NSS. The prevalence of infectious disease was higher among elderly population (aged 60 and above) followed by those aged below 15 years old. Infectious disease was less among adolescent and young population in the age group 15-34. Morbidity related to CVDs and NCDs was extremely higher among the population aged 60, and above as compared to others, it was more so in case of NCDs. Disability and other morbidities were also higher among older population.

There were a minor differences in reporting the prevalence of self-reported morbidity among the caste groups except for CVDs and NCDs. The prevalence of CVDs and NCDs were higher among the other caste group compared to others. The CVDs, NCDs, disability, and other morbidity were higher among Christians as compared to the population of all other religion. Never married women were at a lower risk of all the morbidity. Conversely, widowed or separated women had a higher prevalence of all the five types of morbidities examined. Further, self-reported morbidity by various household wealth quintile did not vary much expect for the morbidity related to CVDs and NCDs. The population from the richest quintile reported higher morbidity compared to others. Almost all the morbidities were higher in Southern region as compared to all other regions (Table 3).

## Results of multivariate analysis

The results of logistic regression are presented in Table 4. Except for infectious disease, females were more likely than males to report self-reported morbidities after controlling for the confounders. Infectious disease, disability, and other morbidities were less likely among population residing in urban areas as compared to rural areas. On the other hand, CVDs and NCDs were more likely among the urban residents as compared to rural residents. Infectious disease was significantly less likely with an increase in education level as compared to people with no education. Conversely, CVDs were more likely among educated group as compared to people with no education. Disability and other morbidities were less likely among the educated population. Although all kinds of morbidities were more likely with an increase in age, it was substantially likely in case of CVDs. Interestingly, all the morbidities were less likely in large families as compared to small families (less than 5 members in a family). All other morbidities except disability in the recent rounds were more likely among OBC and other caste group. The richest MPCE groups were more likely to report all kinds of morbidity as compared to the poorest except for the infectious disease in the second round of NSS. In the first round of NSS, infectious disease was more likely in the north-eastern region however, it was less likely in the subsequent rounds. Moreover, infectious disease was more likely in the western and eastern region in the recent round of NSS. In the first round of NSS, CVDs were more likely in the southern region alone but in the second round, together with southern region, western region was also more likely to report CVDs. Subsequently, in the third round of NSS, eastern region additionally was more likely to report CVDs as compared to northern region. Furthermore, all other regions were less likely to report CVDs as compared to the northern region. In the second round southern region was more likely to report NCDs, but in the third round NCDs were more likely in western, eastern and southern region. Similarly, disability and other morbidities were also more likely in the southern region.

**Table 4:** Adjusted effects of selected background characteristics of self reported morbidities in India, 1995-2014

Background Characteristics	Infectious			CVDs			NCDs			Disability			Others		
	1995	2004	2014	1995	2004	2014	1995	2004	2014	1995	2004	2014	1995	2004	2014
*Sex*															
Male ^a^															
Female	0.891***	0.902***	1.001	1.362***	1.278***	1.196***	1.011	1.021	1.226***	1.001	1.028	1.334***	1.061***	1.111***	1.071**
*Place of Residence*															
Rural ^a^															
Urban	0.890***	0.841***	1.032	1.187**	1.480***	1.387***	1.051	1.03	1.253***	0.834***	0.935**	0.971	0.897***	0.978	1.007
*Education*															
Illiterate ^a^															
Primary	0.646***	0.687***	0.628***	1.471***	1.567***	1.441***	0.852***	0.938**	1.181***	0.949	0.967	1.074**	0.801***	0.654***	0.729***
Higher Secondary	0.612***	0.644***	0.618***	1.387***	1.427***	1.182***	0.753***	0.887***	1.105**	0.724***	0.796***	0.748***	0.768***	0.666***	0.773***
Graduate & above	0.421***	0.476***	0.488***	1.154	1.286***	0.928	0.692***	0.693***	0.943	0.636***	0.554***	0.462***	0.556***	0.475***	0.602***
*Age Group*															
<15 ^a^															
15-34	0.691***	0.685***	0.578***	3.144***	3.139***	0.989	0.981	1.013	1.178***	1.311***	1.78***	3.043***	0.707***	0.595***	0.535***
35-59	1.171***	1.188***	0.829***	23.822***	33.873***	10.777***	2.008***	2.56	5.306***	3.079***	4.413***	9.148***	1.017	0.834***	0.634***
60+	2.233***	2.073***	1.146***	104.348***	160.523***	42.659***	6.756***	8.962	14.413***	13.176***	22.550***	20.526***	1.842***	1.438***	0.798***
HHs Size															
1-5 ^a^															
6-11	0.703***	0.791***	0.673***	0.557***	0.650***	0.705***	0.610***	0.682***	0.691***	0.644***	0.773***	0.684***	0.700***	0.695***	0.670***
>12	0.575***	0.638***	0.548***	0.346***	0.464***	0.479***	0.431***	0.526***	0.477***	0.466***	0.594***	0.619***	0.503	0.548***	0.497***
*Castes*															
ST/SC ^a^															
OBC		1.009	0.902***		1.312***	0.969		1.087**	1.033		1.118**	0.920**		1.152***	1.024
Other	1.125***	1.067	0.97951	1.215**	1.710***	1.201***	1.18	1.240***	1.105**	1.200***	1.272***	1.034	1.095***	1.139***	1.043
*MPCE*															
Poorest ^a^															
Poor	1.174***	0.937	1.049	1.609***	1.344***	1.101***	1.231***	1.092**	1.061	1.014	1.013	1.095**	1.070**	0.983	1.054
Medium	1.243***	0.869***	1.063	2.139***	1.503***	1.255***	1.380***	1.252***	1.170***	1.27***	1.029	1.175***	1.131***	1.088*	1.03
Rich	1.285***	0.915*	1.189***	2.932***	2.182***	1.454***	1.696***	1.403***	1.380***	1.377***	1.042	1.252***	1.219***	1.110**	1.128**
Richest	1.436***	0.913*	1.269***	4.825***	3.0187***	2.005***	2.324***	1.781***	1.835***	1.764***	1.239***	1.417***	1.323***	1.148***	1.208***
*NSS Region*															
North region ^a^															
West region	0.581***	0.792***	1.110***	0.767***	1.436***	1.403***	0.582***	1.003	1.094*	0.823***	1.197***	0.835***	0.623***	0.743***	0.603***
East region	0.794***	0.741***	1.356***	0.851*	0.988	1.362***	0.804***	0.766***	1.306***	0.922	0.747***	1.187***	0.805***	0.799***	1.165***
North-East region	1.132**	0.992	0.522***	0.486***	0.546***	0.226***	0.618***	0.449***	0.167***	0.588***	0.405***	0.273***	0.789***	0.529***	0.403***
South region	0.668***	0.599***	1.376	1.283***	2.242***	3.759***	0.953*	1.460***	2.578***	1.294***	1.465***	1.691***	0.920***	1.140***	1.709***
Central region	0.583***	0.710***	0.954	0.475***	0.921	0.762***	0.463***	0.651***	0.900*	0.437***	0.800***	0.680***	0.764***	0.777***	0.768***

Sources: NSSO Data, 52^nd^, 60^th^ & 71^st^ round,

*p <0.1, **p< 0.05; ***p <0.01

^a^ Reference group for the multivariate logistic analysis

## Discussion

Self-reported morbidities have been on the rise over the last two decades (1995-2014) in all the Indian states. One of the important critiques of the self-reported measure is the reporting bias. Factors such as levels of educational attainment, media exposure, economic status, caste, custom etc. contribute to self-reported bias [9]. However, in the absence of availability of adequate information on morbidity based on medical diagnosis, self-reported morbidity prevalence gives an insight to understand the morbidity profile of the population. The result indicated that self-reported morbidities doubled during the last two decades, of which CVDs increased by almost seven times. Except other morbidities, all other morbidities classified as infectious disease, NCDs, CVDs, and disability increased drastically. The decreasing trend of other morbidities may be due to change in the classification of morbidities in the recent round because, fewer number of morbidities were included in other morbidity category in the recent round as compared to the previous rounds of NSS.

Although infectious diseases are on the rise, a decreasing trend in infectious disease is observed in the rural areas, a situation which can be attributed to better sanitation, awareness and healthcare facilities [16]. On the other hand, infectious disease in urban area is increasing, signaling a serious concern for urban planning and health care provisions in the urban area. Similarly, the rise of CVDs in urban areas is alarming. Due to rising pattern of CVDs and NCDs in the cities [17, 18], it is likely that the cities will be more vulnerable to both communicable and non-communicable disease [19]. The results show more number of females reporting self-reported morbidity compared to their male counterparts, this rise being particularly acute among urban females. Recent study indicates that hypertension was significantly higher among urban females as compared to rural females [20]. Further, the prevalence of morbidity including infectious diseases, CVDs and NCDs were considerably higher among the elderly population [21, 22].

While Kerala, Tamil Nadu, West Bengal, Punjab, undivided Andhra Pradesh remarkably improved in their demographic characteristics, but the incidence of self-reported morbidity is moving parallel upward in these states [22]. Morbidities in Kerala increased by three fold in last two decades, of which CVDs alone increased by 10 times and NCDs increased by 6 times. In addition, infectious disease in Kerala was also quite higher compared to other states. A higher prevalence of NCDs, CVDs, and infectious disease may also be partly because of the presence of a larger percentage of old age population in Kerala. On the other hand, the lifestyle of the socio-economically well-off population in general is one of the important factors responsible for morbidity especially, NCD and CVD [15, 23]. According to the Census of India 2011, Kerala records the highest levels of literacy (95%), in India. It is most likely that due to higher socio-economic status, morbidity reporting is higher in Kerala and other progressive states such as Tamil Nadu, Punjab, West Bengal and undivided Andhra Pradesh [24]. However, literacy rates in most of the north-eastern states are also comparatively higher but self-reported morbidity prevalence on the contrary were much lower. Therefore, high educational status although improves self-reported morbidity yet, may not necessarily increase the prevalence of morbidity. Studies suggest that variations in self-reported morbidity occur because of health ideals, accessibility of health services and the socioeconomic background of the population or it could be due to variation in disease profile between the populations arising from varying levels of demographic and epidemiological transition [22, 25, 26]. Moreover, the burden of self-reporting depends on nutritional status, poverty, female education, working environment, domestic violence, and accessibility to healthcare facilities [27, 28].

The poorer states such as Bihar, Madhya Pradesh and Rajasthan reported a very low level of morbidity. In the earlier rounds of NSS, Assam and Himachal Pradesh reported relatively high levels of morbidity, however, in the recent round, self-reported morbidities in these states were comparatively low. On the other hand, undivided Andhra Pradesh, Odisha, and Tamil Nadu reported low levels of morbidities in the earlier rounds of NSS but indicated an increasing trend in the recent rounds. There is a clear shift in self-reported morbidities from north-eastern States (Tripura & Assam) to south Indian states. In particular, the prevalence of self-reported morbidity rapidly increased in the country’s southern part (Kerala, Tamil Nadu, Andhra Pradesh and Goa). The results of the logistic regression model suggest that sex, place of residence, education, age group, MPCE, caste, marital status, and household size emerges as significant determinants of self-reported morbidity in India. Like many other studies, this study also documents the prevalence of NCDs to be higher among the educated, affluent and urban population [29].

### Limitations

Although this study provides a snapshot of the emerging patterns of self-reported morbidity, covering a span of last two decades from a population-based sample, the findings need to be taken in light of a few limitations. In general, self-reported morbidity may be under-reported [30] but it is also likely over-reported among the health conscious and educated respondents. Study conducted in the past suggests that self-reported morbidity is affected by levels of educational attainment, media exposure, economic status, caste, custom etc. of the respondent [9]. The overall sample size from the 52^nd^ round of NSS (1995) to the most recent round (2014) has considerably declined, as a result it is likely that the prevalence estimates across various rounds of NSS is affected. On the other hand, there have been slight mismatch in the classification of the types of morbidities from 1995-2014 (Appendix). For example, in 2014 (71^st^ round) morbidity schedule introduced ‘all other fevers’ (includes malaria, typhoid and fevers of unknown origin) as other morbidity but, in 2004 morbidity schedule, malaria was categorized under infectious disease. Therefore, it is likely to have affected in the prevalence of self-reported morbidity. Other backward class was included in other caste in the first round of NSS. Hence, a higher prevalence of self-reported morbidity among the other caste group needs to be read in this light. Moreover, food habits, life style, physical activity etc. [31–33] which may have a significant bearing especially on NCDs have not been examined in this study. There is a scope to include these factors in large scale nationally representative survey such as NSS. Despite these limitations, the emerging trend analysis in this study is useful to understand the morbidity conditions in the states of India to inform policy on management of infectious, CVDs, NCDs and disability related morbidities in India.

## Conclusion

Over the years a marginal increase in life expectancy is observed in India. However, increasing prevalence of morbidities in India is a major cause of concern. In this study, Kerala emerged as the leading state with very high prevalence of self-reported morbidities followed by Tamil Nadu, Andhra Pradesh, West Bengal and Punjab across the three rounds of NSS and in all the five broad morbidity categories examined. On the contrary, the poorer states have indicated a lower prevalence rate in most of the morbidities examined in the study. Similarly, north-eastern states like Manipur, Arunachal Pradesh reported a very low prevalence of self-reported morbidity from the very first round of the survey.

Health care provision for NCDs and CVDs at primary level needs to be ensured for early screening and treatment which is almost non-existent at present. Particularly, primary health care for NCDs and CVDs need to be made available in the urban areas. Appropriate policies aimed at the elderly care are the need of the hour. Specialized health care provision for the elderly at the primary level need to be synchronized. In addition, support and care for the elderly from the family members can work as an entity to safeguard in the larger interest of the elderly population. The results reflect that the families constituting of five or more members reported relatively lower levels of morbidity as compared to families having less than five members. There is a greater need for health education for both communicable and non-communicable disease among the population. Health promotion measures may be taken to inform people inculcate healthy habits for prevention of diseases. The health mangers must consider a health facility that is friendly and culturally acceptable for old age population and for the female population. Additionally, health promotion measures may be taken to inform people inculcate healthy habits.

## Appendix

**Table 5:** Classification of disease based on ICD (WHO, 2012)

1995(52^nd^)	2004(60^th^)	2014(71^st^)
Infectious Disease		
*Diarrhoea/ dysentery*	*Diarrhoea/ dysentery*	*Fever with loss of consciousness or altered consciousness*
*Tetanus*	*Gastritis/gastric or peptic ulcer*	*Fever with rash/ eruptive lesions*
*Diphtheria*	*Worm infestation*	*Fever due to Diphtheria, Whooping cough*
*Whooping Cough*	*Amoebiosis*	*Tuberculosis*
*Meningitis and Viral Encephalitis*	*Tuberculosis*	*Filariasis*
*Chicken pox*	*Diseases of skin*	*Tetanus*
*Measles/German Measles*	*Sexually transmitted diseases(STD)*	*HIV/AIDS*
*Mumps*	*Malaria*	*Other sexually transmitted diseases*
*Acute respiratory infection (Including pneumonia)*	*Eruptive*	*Diarrheas/ dysentery etc.*
*Chronic Ameobiosis*	*Mumps*	*Worms infestation*
*Pulmonary Tuberculosis*	*Diphtheria*	*Discomfort/pain in the eye with redness or swellings/ boils*
	*Whooping cough*	*Acute upper respiratory infections (cold, runny nose etc.)*
*Sexually transmitted diseases*	*Tetanus*	*Cough with sputum with or without fever and NOT diagnosed as TB*
*Guinea Worm*	*Filariasis/Elephantiasis*	*Skin infection (boil, abscess, itching)*
*Filariasis (elephantiasis)*		
*gastritis/hyper-acidity gastric/peptic ulcer*		
Cardio Vascular Disease		
*Heart failure*	*Heart disease*	*Stroke/ hemiplegia*
*diseases of heart*	*Hypertension*	*Hypertension*
*high/low blood pressure*		*Heart disease: Chest pain, breathlessness, Cardio-vascular diseases*
Non communicable Disease		
*Cerebral Stroke*	*Hepatitis/Jaundice*	*Jaundice*
*Cough and Acute bronchitis*	*Respiratory including ear*	*Cancer*
*Ailment relating to pregnancy & child birth*	*Bronchial asthma*	*Anaemia (any cause)*
*Jaundice*	*Diseases of kidney/urinary system*	*Bleeding disorders*
*Cancer*	*Prostatic disorders*	*Diabetes*
*Other tumours*	*Gynaecological disorders*	*Under-nutrition*
*(General debility) Anemia*	*Neurological disorders*	*Goitre and other diseases of the thyroid*
*Goitre & thyroid disorders*	*Psychiatric disorders*	*Others (including obesity), High Cholesterol*
*diabetes*	*Conjunctivitis*	*Cataract*
*beri beri*	*Glaucoma*	*Glaucoma*
*rickets*	*Cataract*	*Earache with discharge/bleeding from ear/ infections*
*other malnutrition diseases*	*Goitre*	*Bronchial asthma etc.*
*epilepsy*	*Diabetes mellitus*	*abnormality in urination*
*other diseases of nerves*	*Under-nutrition*	*Pelvic region/reproductive tract infection*
*piles*	*Anaemia*	*Change/irregularity in menstrual cycle*
*diseases of kidney/urinary system*	*Cancer and other tumours*	*Pregnancy with complications before or during labour*
*prostrate disorder*		*Complications in mother after birth of child*
		*Illness in the newborn/ sick newborn*
Disability Disease		
*Diseases of eye*	*Disorders of joints and bones*	*Mental retardation*
*Acute diseases of ear*	*Locomotor*	*Mental disorders*
*Diseases of mouth, teeth and gum*	*Visual including blindness (excluding cataract)*	*Headache*
*Injury due to accident and violence*	*Speech*	*Seizures or known epilepsy*
*mental and behavioural disorder*	*Hearing*	*Weakness in limb muscles and difficulty in movements*
*visual disability (other than cataract)*	*Diseases of Mouth/Teeth/Gum*	*Others including Impaired cognition, memory loss, confusion*
*cataract*	*Accidents/Injuries/Burns/Fractures/Poisoning*	*Decreased vision*
*other diseases of eye*		*Others (including disorders of eye movements)*
*hearing disability*		*Decreased hearing or loss of hearing*
*other diseases of ear*		*Diseases of mouth/teeth/gums*
*speech disability*		*Joint or bone disease/ pain or swelling in any of the joints*
*diseases of mouth, teeth and gum*		*Back or body aches*
*hydrocele*		*Accidental injury, road traffic accidents and falls*
*pains in joints*		*Accidental drowning and submersion*
*other disorder of bones and joints*		*Burns and corrosions*
*locomotor disability*		*Poisoning*
*other congenital deformities (excluding disability)*		*Intentional self-harm*
		*Assault*
Others Disease		
*Fever of Short duration*	*Fever of unknown origin*	*All other fevers(Includes malaria, typhoid and fevers of unknown origin,)*
*other diagnosed ailment (of less than 30 days)*	*Other diagnosed ailments*	*Pain in abdomen: Gastric and peptic ulcers/ acid reflux/ acute abdomen*
*Undiagnosed ailment (of less than 30 days)*	*Other undiagnosed ailments*	*Lump or fluid in abdomen or scrotum*
*other diagnosed ailment (of more than 30 days)*		*Gastrointestinal bleeding*
*Undiagnosed ailment (of more than 30 days)*		*Contact with venomous/harm-causing animals and plants*
		*Symptom not fitting into any of above categories*
		*Could not even state the main symptom*

## Abbreviations

CVDs: Cardio vascular diseases
EAG: Empowered action group
FSU: First stage units
GDP: Gross domestic product
GOI: Government of India
ICD: International classification of disease
MPCE: Monthly per capita expenditure
NCDs: Non communicable diseases
NSSO: National sample survey organization
RGI: Registrar General of India
SC: Scheduled caste
ST: Scheduled tribe
UFS: Urban frame survey
WHO: World Health Organization
